# DNA topoisomerase 1 represses HIV-1 promoter activity through its interaction with a guanine quadruplex present in the LTR sequence

**DOI:** 10.1186/s12977-023-00625-8

**Published:** 2023-05-30

**Authors:** María José Lista, Anne-Caroline Jousset, Mingpan Cheng, Violaine Saint-André, Elouan Perrot, Melissa Rodrigues, Carmelo Di Primo, Danielle Gadelle, Elenia Toccafondi, Emmanuel Segeral, Clarisse Berlioz-Torrent, Stéphane Emiliani, Jean-Louis Mergny, Marc Lavigne

**Affiliations:** 1 Université Paris Cité, Institut Cochin, INSERM, CNRS, F-75014 Paris, France; 2 CNRS UMR 5320, INSERM U1212, ARNA, Univ. Bordeaux, IECB, 33000 Bordeaux, France; 3 Institut Pasteur, Bioinformatics and Biostatistics Hub, Université Paris Cité, 75015 Paris, France; 4 Present Address: Institut Pasteur, Departement of Virology, Université Paris Cité, 75015 Paris, France; 5 Institut de Biologie Integrative de la Cellule, CNRS, Université Paris-Saclay, 91198 Gif Sur Yvette, Cedex, France; 6 Laboratoire d’Optique et Biosciences, Ecole Polytechnique, CNRS, INSERM, Institut Polytechnique de Paris, 91120 Palaiseau, France; 7 Present Address: Department of Infectious Diseases, School of Immunology and Microbial Sciences, King’s College London, London, UK; 8 Present Address: Université de Strasbourg, CNRS UPR 9002, Architecture et réactivité de l’ARN, 67000 Strasbourg, France; 9 Present Address: School of Engineering, China Pharmaceutical University, Nanjing, 211198 China

**Keywords:** Guanine quadruplex, HIV-1 LTR promoter, HIV-1 transcription, HIV-1 latency, DNA topoisomerases, Transcriptional regulation, Host-virus interaction

## Abstract

**Background:**

Once integrated in the genome of infected cells, HIV-1 provirus is transcribed by the cellular transcription machinery. This process is regulated by both viral and cellular factors, which are necessary for an efficient viral replication as well as for the setting up of viral latency, leading to a repressed transcription of the integrated provirus.

**Results:**

In this study, we examined the role of two parameters in HIV-1 LTR promoter activity. We identified DNA topoisomerase1 (TOP1) to be a potent repressor of this promoter and linked this repression to its catalytic domain. Additionally, we confirmed the folding of a Guanine quadruplex (G4) structure in the HIV-1 promoter and its repressive effect. We demonstrated a direct interaction between TOP1 and this G4 structure, providing evidence of a functional relationship between the two repressive elements. Mutations abolishing G4 folding affected TOP1/G4 interaction and hindered G4-dependent inhibition of TOP1 catalytic activity in vitro. As a result, HIV-1 promoter activity was reactivated in a native chromatin environment. Lastly, we noticed an enrichment of predicted G4 sequences in the promoter of TOP1-repressed cellular genes.

**Conclusions:**

Our results demonstrate the formation of a TOP1/G4 complex on the HIV-1 LTR promoter and its repressive effect on the promoter activity. They reveal the existence of a new mechanism of TOP1/G4-dependent transcriptional repression conserved between viral and human genes. This mechanism contrasts with the known property of TOP1 as global transcriptional activator and offers new perspectives for anti-cancer and anti-viral strategies.

**Supplementary Information:**

The online version contains supplementary material available at 10.1186/s12977-023-00625-8.

## Background

More than 35 million people are infected by HIV-1 and half of them receive Highly Active Antiretroviral Therapies (HAART), which target different steps in the viral replication cycle. Although these therapies efficiently block HIV-1 propagation, they are not effective at completely eliminating the infection. This is due to the presence of “silent” unexpressed copies of the virus that remain undetectable in the patient. This condition is known as viral latency [1]. These latent viral reservoirs can last for a long period of time with only a few copies of replication-competent viruses that are the main source of viral rebound upon HAART interruption. The mechanisms regulating viral gene expression are closely related to the process of viral latency [2, 3]. Therefore, understanding these mechanisms could help in developing effective therapeutic strategies to completely eradicate viruses in infected individuals [1, 4].

Once integrated into the cell genome, HIV-1 uses the cellular RNA polymerase II (RNA Pol II) transcription machinery to transcribe its own genome [5]. This machinery recognizes the promoter located in the 5′ long terminal repeat (5′-LTR) of the viral genome and its activity is regulated by viral and cellular proteins. Some of these proteins, such as SP1 or NF-κB, interact directly with specific sites located upstream of the transcriptional start site (TSS) and regulate the initiation step. Several other regulators act on the chromatin landscape covering the viral genome and regulate the initiation, elongation, or termination steps [6]. The genomic and nuclear locations of the integrated genome also contribute to HIV-1 transcriptional regulation, either through transcriptional interference between viral and neighbor cellular promoters or through the existence of active or repressive environments surrounding the integrated genome [7, 8].

DNA topoisomerases (DNA Topos) are essential to solve the DNA topological constraints generated by different processes occurring on the cell genome, such as DNA replication, transcription or DNA repair (reviewed in [9, 10]). Their activities require the cleavage of the target DNA by a catalytic tyrosine of the enzyme (Y723 in human TOP1). Topoisomerases are classified as type I or type II, depending on their ability to cleave only one DNA strand (type I enzymes, such as TOP1 and TOP3) or the two strands of the DNA helix (type II enzymes, such as TOP2α and TOP2β). DNA Topos regulate transcription and translation of several genes and the chromatin dynamics along these genes [11–13]. They can be distinguished by their gene specificities and regulatory mechanisms. TOP1 is the primary relaxer of DNA torsion constraints at genes exhibiting low or intermediate levels of transcription [11]. It is involved in the activation of inflammatory genes after bacterial or viral infections [14]. Furthermore, it interacts with the phosphorylated form of RNA Pol II which is enriched in the elongated part of the transcribed genes [15]. This interaction stimulates TOP1 catalytic activity, facilitating the release of topological constraints both upstream and downstream of the transcription machinery. DNA Topo activities are also linked with chromatin dynamics and modifications. The topological constraints regulated by these enzymes during transcription elongation help to maintain the stability of the nucleosomes, both upstream and downstream of the transcription machinery [13]. In addition, nucleosome remodelers, such as BRG1/SMARCA4, can be involved in the recruitment of TOP1 or TOP2 to active or repressed chromatin domains [16, 17]. Finally, TOP1 and TOP2A can act in concert to regulate transcription, as recently shown on cellular genes regulated by MYC complexes [18].

The mechanisms of transcriptional regulation of cellular genes by DNA Topos are widely investigated. However, the exact role of these enzymes as regulators of HIV-1 transcription is still not well understood. Camptothecin and Topotecan, two TOP1 poison inhibitors, are able to repress viral transcription in a TOP1-independent manner [19–21]. Moreover, TOP2B can activate HIV-1 transcription, and this effect is reliant on the presence of the viral TAT protein [22]. The presence of a pause of the RNA Pol II machinery after the TAR sequence and its release by TAT make HIV-1 transcription a perfect model to study transcriptional regulation by DNA topoisomerases.

G-quadruplexes (G4) are DNA or RNA secondary structures folded in G-rich regions and formed by the stacking of two or more guanine quartets stabilized by a central spine of cations, typically potassium [23]. These structures are highly polymorphic and have been extensively studied in vitro, using several biophysical and structural approaches [24]. In the human genome, ChIP-seq studies have revealed hundreds of thousands of G4s, and their enrichment in nucleosome-depleted regions, promoters and 5′UTR of highly transcribed genes [25, 26]. Algorithms have also been developed to predict G4 propensity at the genome-wide level [27, 28]. One of them, G4hunter, allows to predict putative G4 forming sequence (PQS) with unprecedented accuracy [27, 29]. This algorithm provides a propensity score instead of a Yes/No answer, allowing to choose a threshold relevant to each case.

TOP1 physically interacts with G4 structures [30–33] and this interaction is associated with an inhibition of its catalytic properties [32–34]. In *S. cerevisiae*, *Top1* depletion or silencing favors the formation of G4s, and this effect results from an increased negative topology [35]. TOP1 depletion or its replacement by TOP1 catalytic mutants are also responsible for an increase in genomic instability of G4-rich transcribed sequences [36, 37]. This functional link between TOP1 and G4s is reinforced by the identification of *Top1* as a major G4 ligand sensitizer gene. This was shown by three different shRNA screens looking for genes increasing the sensitivity of cancer cells to G4 ligands [38]. These different observations support a strong genetic interaction between the *Top1* gene and G4 structures, with high therapeutic impact [39]. However, the biochemical, structural and functional determinants of this interaction are still poorly documented.

G4s regulate the expression of several human genes, especially oncogenes, and the mechanisms and partners of these regulations are very diverse [40, 41]. At the DNA level, G4s present upstream of the TSS can recruit transcription factors, such as SP1, that will regulate the formation of the transcription initiation machinery [42, 43]. G4s can also act on the chromatin structure, either by evicting nucleosomes, by recruiting histone modifying or nucleosome remodeling complexes [44] or by interfering with the formation of topological associated domains and DNA loops between enhancer and promoter sequences. Conversely, the folding of G4 present in active promoters is regulated by chromatin structure and nucleosome depletion of the G4 locus [45].

G4s play an important role in the regulation of the replication of several viruses and a large part of these regulations occurs at the transcriptional level [46–48]. HIV-1 provides a well-studied example of transcriptional regulation by DNA G4s. While G4s are mainly involved in activation of eukaryotic cells transcription, G4s present in the HIV-1 promoter mainly repress viral transcription [49, 50]. Indeed, the U3 region of the HIV-1 LTR contains several PQSs, located 105 to 48 nucleotides upstream of the TSS [51–55]. This location is highly conserved between retroviruses [49, 56] and corresponds to a nucleosome-free region of the viral promoter. In vitro, the formation of G4 structures with oligonucleotides covering these sequences has been demonstrated [52–54]. In cells, some of these PQS, such as the LTR III sequence, are involved in transcriptional repression of the viral promoter [50]. In addition, several cellular proteins, such as Nucleolin, hnRNPA2/B1 and FUS, have been shown to bind to these PQS and to modulate viral transcription [55, 57, 58]. Since transcription is an essential step of HIV-1 replication, G4s are new targets for anti-viral strategies and some G4 ligands or mimicking oligonucleotides have already shown promising anti-viral activities [52, 59–61].

In this study, we examined the effects of human DNA TOP1, TOP2A and TOP2B on the transcription of HIV-1. We discovered that TOP1 represses viral promoter activity, with its catalytic activity playing an important role in this repression. No effect of TOP2 enzymes on the viral transcription was observed. We also demonstrated a direct interaction between TOP1 and a G4 motif present in the HIV-1 LTR promoter. Moreover, mutations abolishing G4 folding affected TOP1 binding and induced a reactivation of transcription starting at the viral promoter. Finally, we revealed an enrichment of predicted G4 structures in the promoter of the TOP1-repressed cellular genes. Our findings reveal a novel TOP1-G4 dependent transcriptional repression mechanism, which could be used in anti-viral or anti-cancer strategies.

## Results

### TOP1 represses HIV-1 LTR promoter activity, and this repression depends on TOP1 catalytic activity

**Fig. 1:**
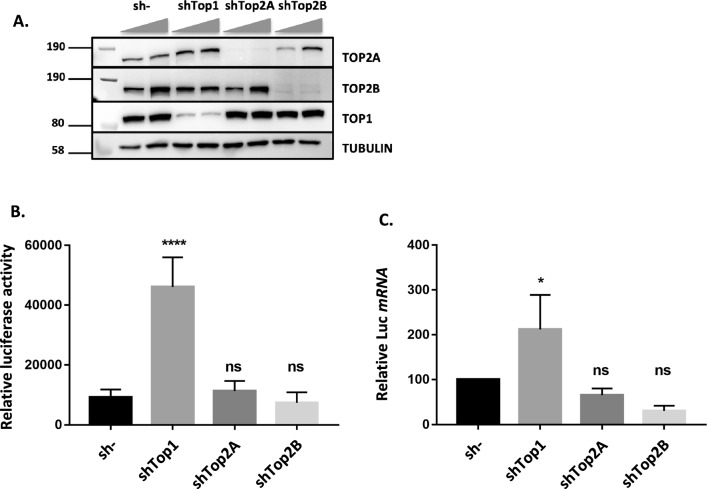
Top1 shRNA silencing induces a reactivation of HIV-1 LTR promoter activity in HeLa-LTR luciferase cells. HeLa LTR luciferase cells were transduced with pLKO.1 vector expressing shRNAs directed against *Top1*, *Top2A* or *Top2B* genes or a scrambled shRNA sequence (sh-), at ratios of 100–300 µL vector/2. 10^6^ cells. **A** The amount of TOP1, TOP2A and TOP2B proteins was evaluated 7 days post-transduction by SDS-PAGE and Western-Blot of cellular extracts. The Luciferase activity (**B**) or the levels of mRNA coding for the *luciferase* gene (**C**) were also quantified 7 days post-transduction (n = 5) at the highest transduction ratio

HeLa LTR luciferase cells have been established to study HIV-1 transcriptional regulation [62]. Their genome contains a unique copy of the *Luciferase* gene under the control of the HIV-1 LTR promoter. We used these cells to assess the role of three cellular DNA topoisomerases as potential regulators of HIV-1 transcription. *Top1*, *Top2A* and *Top2B* genes were silenced using specific shRNA lentiviral vectors and this silencing was confirmed by a decreased amount of the corresponding enzymes in total cellular extracts, 7 days post-transduction of shRNA lentiviral vectors (Fig. 1A). Both luciferase activity and mRNA levels, measured in these cells, revealed a significant increase of transcriptional activity when *Top1* is silenced, but not when *Top2A* or *Top2B* are silenced (Fig. 1B, C). These results indicate that TOP1 represses HIV-1 LTR promoter activity, and that this repression is specific for this DNA topoisomerase.

To evaluate the conservation of this effect in lymphocytes T cells, we performed a similar topoisomerase silencing strategy in J-Lat cells. These cell lines are derived from Jurkat T-cells and contain a unique HIV-1 construct integrated in their genome, with the GFP gene under the control of the HIV-1 LTR [63]. We chose two different J-Lat clones (A1 and 10.6) which differ by the length of integrated HIV genome (short for A1 and nearly complete for 10.6) and their integration site. Both J-Lat A1 and 10.6 cells are characterized by a repressed HIV-1 transcription, which can be reactivated in the presence of HIV-1 transcription inducers. These cells also differ from HeLa LTR luciferase cells by the presence of the Tat coding sequence in the HIV-1 genome, which allows to evaluate the role of this transcriptional regulator in the observed effect.

**Fig. 2:**
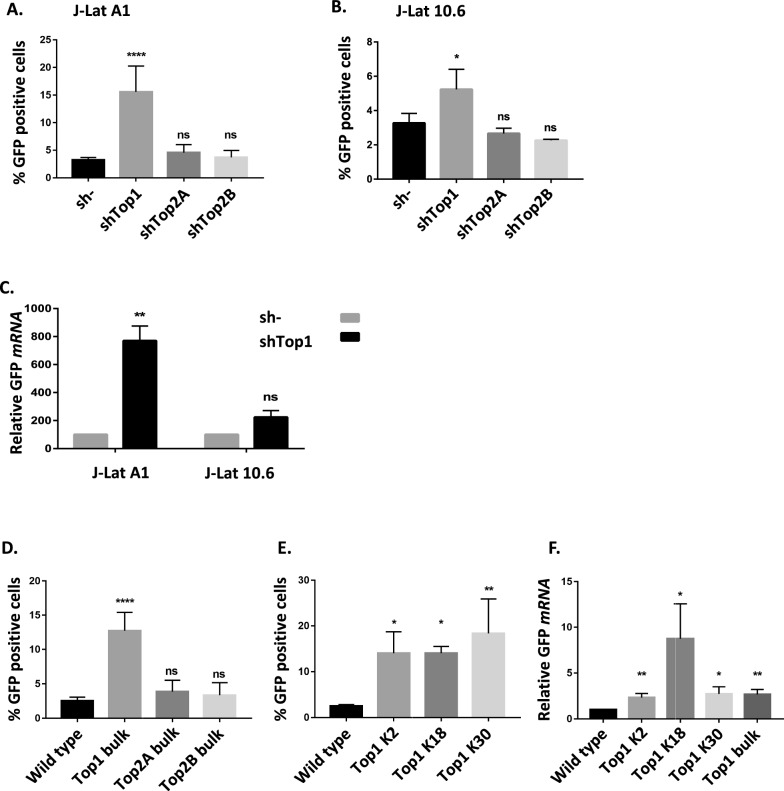
Top1 shRNA silencing or CRISPR/Cas9 depletion induces a reactivation of HIV-1 LTR promoter activity in J-Lat cells. **A**–**C** J-Lat A1 and J-Lat 10.6 cells were transduced with pLKO.1 vectors expressing a shRNA directed against *Top1*, *Top2A* or *Top2B* genes or a scrambled shRNA sequence (sh-) (transduction ratio of 300 µL vector / 2 × 10^6^ cells). The % of GFP positive cells (n = 6 for J-Lat A1 and n = 3 for J-Lat 10.6) (**A**, **B**) or the level of mRNA coding for GFP (n = 3 for both cell lines) **(C)** were quantified 7 days post-transduction. **D**–**F** J-Lat A1 cells were transfected by a LentiCRISPRV2 plasmid expressing guide RNAs directed against *Top1*, *Top2A* or *Top2B* genes. The percentage of GFP-positive cells was measured after 10 days of puromycin selection (n = 9 for WT, n = 5 for Top1 bulk and n = 3 for TopA and Top2B bulk) (**D**). Three clones (K2, K18 and K30) were selected after transfection by a LentiCRISPRV2 targeting Top1 and analyzed for the % of GFP positive cells (n = 3) (**E**) or the level of mRNA coding for GFP (n = 3) (**F**)

Topoisomerases shRNA silencing performed in J-Lat cells is efficient (as shown in Additional file 1: Fig S1A for J-Lat A1) and *Top1* silencing induces a reactivation of HIV-1 LTR promoter activity, in both J-Lat A1 and 10.6 cells as reported by the % of GFP-positive cells measured 7 days post-transduction (Fig. 2A, B). This reactivation is specific for *Top1* and not observed after silencing of *Top2A* or *Top2B* genes. The levels of GFP mRNA measured at the same time confirm this reactivation and indicate that it occurs at the level of transcription (Fig. 2C). Interestingly, reactivation of HIV-1 promoter after *Top1* silencing is lower in J-Lat 10.6 than A1 cells but still significant when the % of GFP cells is measured (Fig. 2B). This difference observed between J-Lat A1 and 10.6 cells could result from the length of the transcribed sequence and the known activation of transcription elongation by TOP1 [15]. This effect would minor the observed repressive effect of TOP1 on transcription initiation.

In addition, we performed CRISPR/Cas9 edition of *Top1*, *Top2A* and *Top2B* genes and studied the consequences on viral transcription. J-Lat A1 cells transfected by a specific lentiviral CRISPRv2 vector targeting each Topoisomerase gene were selected during 10 days in the presence of puromycin. After selection, the efficiency of knockout and the % of reactivated cells were measured by western blot (Additional file 1: Fig. S1B) and flow cytometry (Fig. 2D). The knockout of the three topoisomerase genes was efficient and revealed a significant reactivation of HIV-1 promoter only in *Top1* edited cells. Individual clones, selected from the population of *Top1* CRISPR/Cas9 edited cells, were studied for the amounts of TOP1 and for the corresponding levels of GFP mRNAs and proteins. These values are reported for three representative clones (K2, K18 and K30) (Fig. 2E, F, Additional file 1: Fig. S1C, D), showing that *Top1* edition is associated with an increased HIV-1 LTR expression, measured at both mRNA and protein levels.

**Fig. 3:**
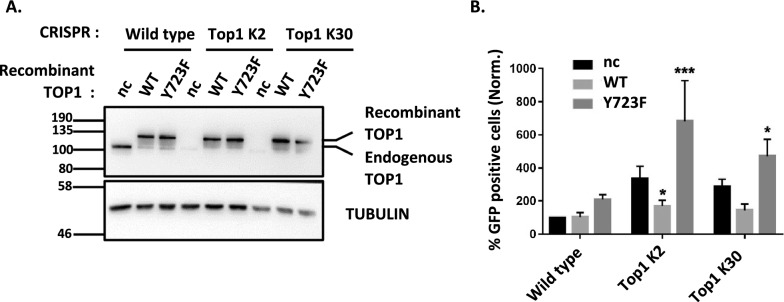
Catalytic active TOP1 restores the repression of HIV-1 LTR promoter in *Top1* depleted J-Lat A1 cells. WT or two CRISPR TOP1 clones of J-Lat A1 cells were transduced by a pTRIP vector expressing WT or Y723F TOP1 or no protein (nc). **A** Seven days post-transduction, the presence of recombinant and endogenous TOP1 proteins was evaluated by SDS-PAGE and western blot directed against TOP1. **B** Concomitantly, the % of GFP-positive cells was measured by FACS and normalized to the % measured in WT cells, transfected by an empty vector (n = 4 for empty vector and n = 3 for expressed protein conditions)

Therefore, both shRNA and CRISPR/Cas9 strategies targeting *Top1*, *Top2A* or *Top2B* genes reveal a novel role of TOP1 as a repressor of HIV-1 LTR promoter in lymphocyte T-cells. To confirm this role, we expressed a wild type (WT) or a catalytic mutant (Y723F) form of this enzyme in WT J-Lat A1 cells and in two CRISPR/Cas9 *Top1* edited clones (K2 and K30). Recombinant proteins contain an N-terminal Tag of 110 amino acids made of a Flag epitope and an Auxin Induced Degron (AID) sequence allowing to differentiate them from the endogenous protein by electrophoretic migration (Fig. 3A). Experimental conditions were optimized to obtain similar levels of recombinant and endogenous proteins.

In J-Lat A1 cells, expression of recombinant WT TOP1 is associated with a lower level of endogenous protein. This replacement of endogenous TOP1 by the WT recombinant construct does not perturb HIV-1 promoter activity, as evidenced by the similar percentages of GFP positive cells (Fig. 3B). Expression of Y723F TOP1 is also associated with a decreased level of endogenous protein. The replacement of WT endogenous TOP1 by this Y723F form induces a slight and non-significant increase of the viral promoter activity.

In TOP1 CRISPR/Cas9 edited clones (K2 and K30), expression of WT recombinant TOP1 restores the repression of HIV-1 promoter activity to levels comparable to WT cells. Interestingly, expression of Y723F TOP1 cannot restore this repression and is even associated with a significant increase in the percentage of GFP-positive cells. These results confirm the role for a catalytic active TOP1 in the repression of HIV-1 promoter activity.

### TOP1 interacts with a G4 present in the HIV-1 promoter and this interaction inhibits TOP1 DNA relaxation activity

The U3 region of the HIV-1 LTR contains several potential quadruplex sequences (PQS) and some of them, such as the LTR III sequence, are involved in transcriptional repression of the viral promoter [50]. In addition, human TOP1 has the ability to interact with G4 structures [30, 32]. Since both TOP1 and HIV-1 U3 PQSs are involved in HIV-1 transcriptional repression, we wondered if this repression could be mediated by a functional interaction between these two elements.

**Fig. 4:**
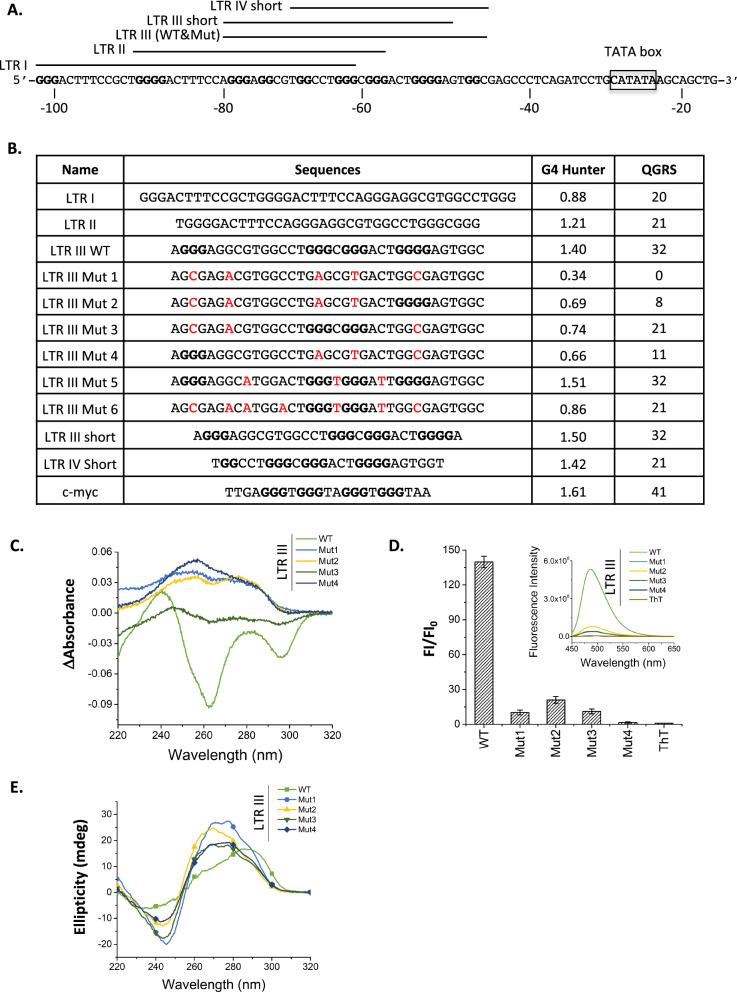
Characterization of G4 structures present in HIV-1 LTR. **A** HIV-1 promoter sequence and previously characterized G4s (LTR I to IV [50, 53, 55]) present along this sequence. **B** Oligonucleotides covering HIV-1 and c-myc G4s used in this study. Name, sequence and G4 folding scores (G4hunter and QGRS) of oligonucleotides covering PQSs present in HIV-1 LTR (LTR I, LTR II and LTR III, LTR III Short and LTR IV Short), mutated PQS LTR III (Mut1 to Mut6) and a PQS present in c-myc promoter. These oligonucleotides were used in the biophysical and structural studies presented below. **C**–**F** Biophysical and structural characterization of WT and mutated HIV-1 LTR III oligonucleotides G4 sequences. **C** Isothermal difference spectra with and without 100 mM KCl. **D** Thioflavin (ThT) fluorescence assay. **E** Circular dichroism (CD) spectra measured in 100 mM KCl. Oligonucleotides used in these studies are described in Additional file 5: Table S1

To determine how TOP1/G4 interaction occurs within the HIV-1 LTR promoter, we selected four PQS found in the U3 region of HIV-1 LTR, defined as LTR I, LTR II and LTR III in [50] and LTR IV in [53] (Fig. 4A, B). Among them, the two LTR III sequences (WT and short) show the highest G4Hunter scores (1.4 and 1.5) [27], in agreement with previous studies on LTR III G4 structure. Please note that the two LTR III sequences used in our study slightly differ from the LTR III sequences used in previously published studies ([50] and [53]). We also designed several mutations of this sequence to prevent its G4 folding (Mut1 to Mut4) or to decrease its GC content (Mut5 to Mut6). G4Hunter allowed us to rank these sequences according to their G4 folding propensity (Mut5 > Mut6 > Mut3 > Mut2 > Mut4 > Mut1, from highest to lowest). As a positive control able to adopt a G4 structure, we selected the PQS present in the c-myc promoter which exhibits a high G4Hunter score (1.61).

Oligonucleotides covering the selected HIV-1 LTR PQSs and the mutated LTR III sequence were first studied in vitro for their potential to fold into a G4 structure using several biophysical and structural approaches (Fig. 4C–F and Additional file 2: Fig. S2). To do so, we collected the isothermal difference spectra (IDS) measured with and without 100 mM KCl (Fig. 4C and Additional file 2: Fig. S2A) and performed a Thioflavin (ThT) fluorescence light-up assay (Fig. 4D and Additional file 2: Fig. S2B), In the ThT assay, we compare the fluorescence emission of ThT alone or in the presence of a candidate sequence. As previously shown, we found an increase in fluorescence when the sequence is forming a quadruplex structure [64]. Circular dichroism (CD) spectra (Fig. 4E and Additional file 2: Fig. S2C) with positive peaks at 295 and 270 nm and a negative peak at 240 nm of LTR III WT shows that it could fold into a stable G4 with hybrid conformation. In addition, complex proton signals in 14–10 ppm range of 1H NMR spectra (Additional file 2: Fig. S2D) confirmed G4 formation for the LTR III WT nucleotide, while the mutated sequences with a lower G4Hunter score (< 1) have a lower propensity to form these structures.

**Fig. 5:**
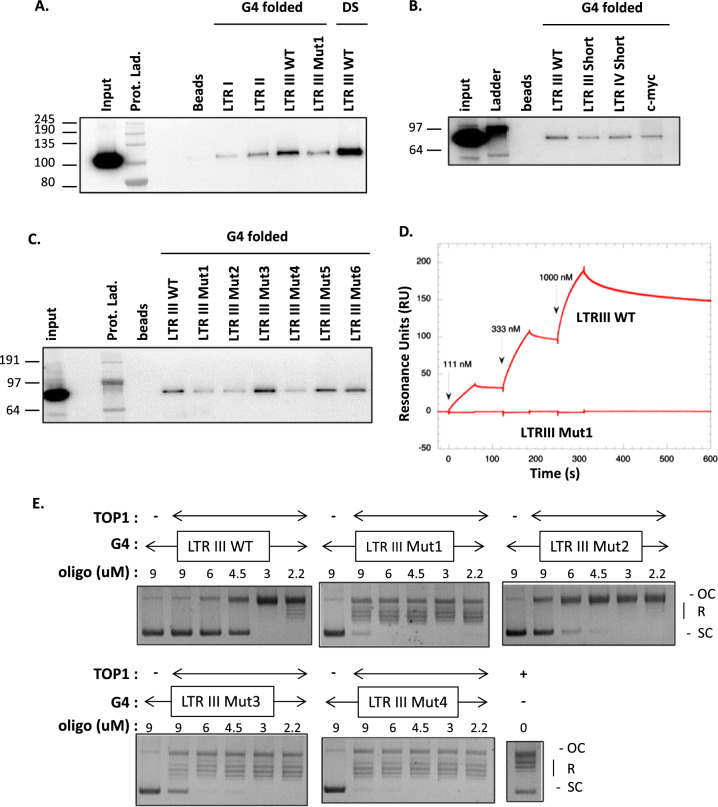
TOP1 interacts with G4 LTR III and this interaction inhibits TOP1 DNA relaxation activity. **A**–**C** G4 DNA pull-down assays performed with biotinylated oligonucleotides covering PQS present in HIV-1 LTR or c-myc promoters (see Fig. 4B and Additional file 5: Table S1). These oligonucleotides, either folded into a G-quadruplex (G4 folded) or hybridized to their complementary strand (DS) were attached to streptavidin magnetic beads and incubated with recombinant human 6His-TOP1 enzyme (**A**) or Jurkat cellular extracts (**B**, **C**). After several washes with increased salinity buffers, retained TOP1 was quantified by SDS-PAGE and western blotting. All these experiments were repeated at least 3 times. **D** Kinetic analysis by SPR of LTR III DNAs binding to TOP1. G4 LTR III WT and Mut1 DNAs, prepared in the running buffer containing 50 mM potassium chloride, were injected at increasing concentrations (111, 333 and 1000 nM) over the protein immobilized by amine coupling. Six independent experiments were performed with DNA samples injected in duplicate. Red lines represent the recorded sensorgrams. **E** Effect of WT and mutated G4 LTR III on TOP1 catalytic activity. This activity was measured by a DNA relaxation assay performed with recombinant human TOP1 (70 nM), a supercoiled pBR322 plasmid (200 ng) and different concentrations of G4-folded LTR III oligonucleotides (2.2 to 9 μM, from right to left). At the end of the reaction, the Open Circular (OC), Relaxed (R) and Supercoiled (SC) forms of the plasmid were separated by electrophoretic migration on a 1% Agarose gel and stained with Ethidium Bromide

Based on these results, oligonucleotides corresponding to the WT and mutated LTR sequences were then studied for their interaction with TOP1. In vitro, we used a pull-down assay based on the attachment of G4-folded biotinylated oligonucleotides covering the studied sequences to streptavidin-coated magnetic beads (Fig. 5A–C). Either human recombinant TOP1 or Jurkat cells extracts were incubated with these beads and after several washes with buffers of increasing salinity, the retained TOP1 fraction was quantified by PAGE and immuno-detection. As shown in Fig. 5A, recombinant TOP1 interacts more strongly with the LTR III G4 than with other G4s formed on the LTR I and LTR II sequences. In addition, TOP1 interacts with the double-stranded (DS) form of LTR III sequence as expected for this enzyme which is known to bind dsDNA. Finally, the loss of G4 structure in Mut1 LTR III (Fig. 4E, F) is associated with a large decrease of TOP1 interaction (Fig. 5A).

We repeated this assay using Jurkat cell extracts (containing natural endogenous TOP1) and oligonucleotides covering HIV-1 LTR and c-myc promoters (Fig. 5B, C) and previously characterized for their G4-folding properties (Fig. 4). These assays confirmed the preferential interaction of TOP1 to oligonucleotides with high G4 folding capacities such as LTR III (WT and short), LTR VI short and c-myc (Figs. 4B and 5B). Among the LTR III mutated oligonucleotides, TOP1 interacts less efficiently with Mut1, Mut2 and Mut4 LTR III sequences, which correspond to the lowest G4 folding capacities. Conversely, Mut3, Mut5 and Mut6 LTR III oligonucleotides, which keeps TOP1 interaction (in pull-down assays), have higher G4Hunter scores (above 0.7) than the other LTR III mutated sequences (Figs. 4C and 5C). Altogether these results demonstrate a specific interaction of TOP1 for the LTR III G4 structure present in HIV-1 promoter and not with any sequence with a high GC content.

In addition, we expressed the WT or Y723F forms of TOP1 in these cells, similarly as in J-Lat A1 cells for complementation assays (Fig. 3B) and total cell extracts were used in pull-down assays on LTR III WT and Mut1 G4 folded oligonucleotides. As shown in Additional file 3: Fig S3., both recombinant and endogenous TOP1 interact with the WT G4 and the Y723F mutation does not affect the interaction of the recombinant enzyme. In addition, the Mut1 sequence preventing G4 folding inhibits the interaction of both WT and Y723F forms of TOP1. Therefore, in our assay, mutating the catalytic site of TOP1 does not affect its preferential interaction to the G4 structure folded on the HIV-1 LTR III sequence.

TOP1/HIV-1 LTR III G4 interaction was also investigated by SPR (Fig. 5D). The G4 forming oligonucleotide and its mutated control sequence designed not to form a G4 were injected at increasing concentrations over the protein immobilized by amine coupling onto the sensor chip surface. Only LTR III WT gave signals increasing in a dose-dependent manner. K_D_ values could not be obtained from the fit of these sensorgrams with a 1:1 Langmuir model of interaction. However, the obtained sensorgrams clearly show that the G4 folding of the LTR III sequence is responsible for its interaction with DNA TOP1.

G4 structures can inhibit TOP1 catalytic activity [33, 34]. We wondered if this property is conserved in the HIV-1 LTR III G4 structure. Using a plasmid relaxation assay and recombinant human TOP1, we tested the effect of LTR III WT G4 on TOP1 catalytic activity and observed a clear inhibition at 4.5, 6 and 9 µM of G4 (Fig. 5E). This inhibition was then tested with four mutated LTR III G4, previously characterized for their lower G4 folding capacities (Mut1 to Mut4) and their decreased affinity for TOP1 (Mut1, Mut2 and Mut4). All of these mutations abolish or significantly decrease the capacity of the G4 oligonucleotides to inhibit TOP1 catalytic activity (until 10 µM for Mut1 and Mut4 and 6 µM for Mut2 and Mut3) (Fig. 5E**)**. Altogether, these results showed that the G4 folded on the HIV-1 LTR III sequence can inhibit TOP1 catalytic activity in vitro and that this inhibition is linked with the G4 structure.

### TOP1-dependent repression of HIV-1 LTR promoter relies on the presence of G4 motives upstream of the TSS

**Fig. 6:**
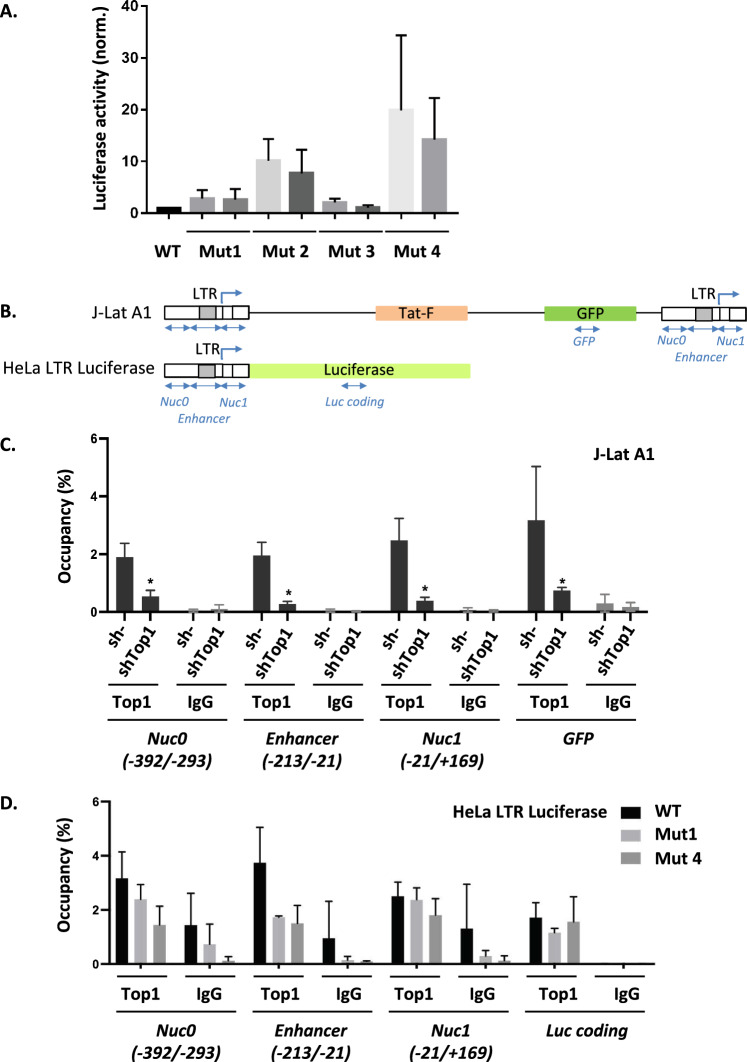
TOP1-dependent repression of HIV-1 LTR promoter activity requires LTR III G4 folding and TOP1 interaction to this G4 structure. **A** Mutations disrupting G4 folding and TOP1 interaction reactivate HIV-1 promoter activity. The luciferase activities of HeLa LTR luciferase clones containing WT or mutated HIV-1 LTR upstream of the *luciferase* gene were measured and normalized for the activity of the WT clone (n = 3). **B** Scheme of the LTR HIV-1 promoter and GFP/Luciferase transcribed genes present in the J-Lat A1 and HeLa LTR Luciferase cell genomes. This scheme highlights the G4 region in the LTR (grey box) and the regions studied by TOP1 ChIP (Nuc0, Enhancer, Nuc1, GFP and Luc coding). **C** TOP1 occupancy along the HIV-1 promoter (Nuc1, Enhancer and Nuc0 regions) and transcribed gene (GFP region) present in the HIV-1 minigenome integrated in J-Lat A1 cells [63]. The occupancy was measured by ChIP assay (n = 3) performed in cells with *Top1* gene previously silenced (shTop1) or control (sh-) (similar conditions as in Fig. 2). **D** TOP1 occupancy along the HIV-1 promoter (Nuc1, Enhancer and Nuc0 regions) and the transcribed gene (Luciferase coding region) measured by ChIP assay (n = 3) performed in HeLa LTR luciferase cells of WT, Mut1 or Mut4 LTR III sequence [62]

If TOP1-dependent repression of HIV-1 promoter depends on its ability to interact with a G4 structure present in the promoter, mutations preventing the folding of this structure should reactivate HIV-1 transcription. We tested this hypothesis by introducing the four sets of mutations described above in the LTR III sequence of the HeLa LTR-luciferase cells. These mutations were introduced in the parental pcDNA5/FRT/LTRHIV-Luciferase plasmid and inserted at the same unique genomic position in clone9 HeLa Flp-In™ cells. For each mutation, two clones containing the same copy number of inserted sequences were selected and the luciferase activity was measured and normalized for the clone containing the WT sequence. This study reveals a reactivation of HIV-1 promoter activity on Mut1, Mut2 and Mut4 LTR III sequences (Fig. 6A), which are the ones with a lower G4 folding potential (Fig. 4C–E and Additional file 2: Fig. S2D) and a decreased TOP1-G4 interaction (Fig. 5C). Taken together, our results show that TOP1-dependent repression requires LTR III G4 folding and TOP1 interaction with this G4 structure.

Finally, we investigated TOP1 binding to the HIV-1 LTR III G4 in a chromatin environment. This study was performed by TOP1 chromatin immunoprecipitation (ChIP) in J-Lat A1 and HeLa LTR Luciferase cells, previously used for transcription studies (Figs. 1, 2). In both cells, the G4 structure is located the enhancer region (scheme of the studied sequence presented in Fig. 6B). In J-Lat A1 cells, we observed a homogenous TOP1 occupancy along the different tested regions in the HIV-1 promoter (Nuc0, Enhancer and Nuc1) and GFP transcribed sequence (Fig. 6C). This result is consistent with the reported interaction of total fraction of TOP1 for both gene promoters and transcribed sequences [15]. To assess the specificity of the interaction between TOP1 and LTR III G4, we performed a similar ChIP study in HeLa LTR-luciferase cells with WT, Mut1 and Mut4 LTR III sequences. Mut1 and Mut4, were already characterized in vitro for their effect on G4 folding (Fig. 4B) and TOP1 interaction (Fig. 5C), but also for their effect on transcription (Fig. 6A). Like in J-Lat A1 cells, TOP1 occupancy is homogenous along the promoter (Nuc0, Enhancer and Nuc1) and transcribed (Luc coding) regions of WT sequence. However, this occupancy is decreased in the enhancer region of both Mut1 and Mut4 sequences but not in the other studied regions. This result demonstrates that TOP1 occupancy in the enhancer region reflects the specific interaction of this enzyme for G4 structures present in this region.

**Fig. 7:**
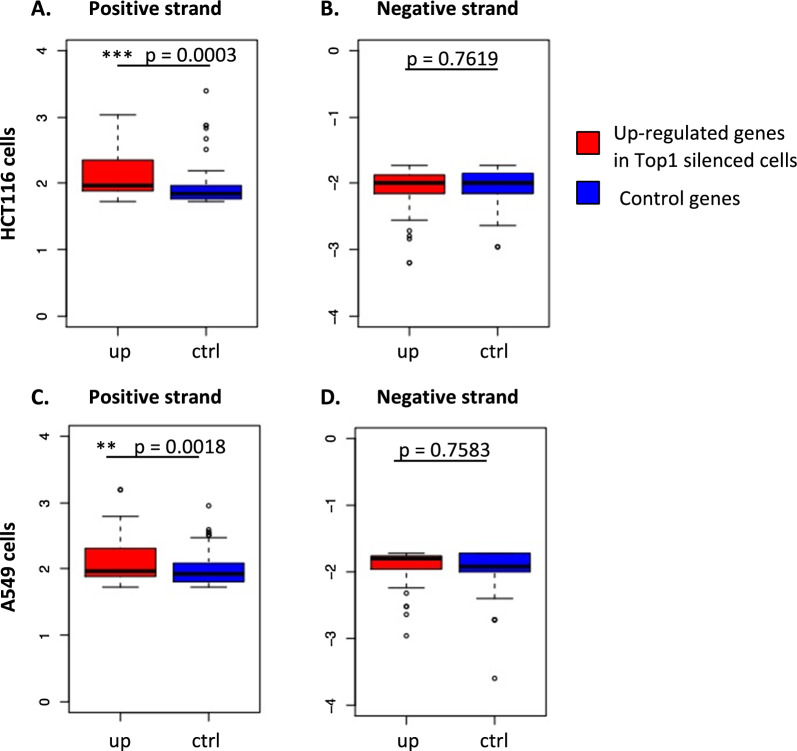
G4 are enriched in the positive strand of TOP1 repressed cellular genes. **A**, **B** Boxplots of G4Hunter maximum scores (score threshold > 1.7, window size = 25 nts) in the positive (**A**) or negative (**B**) strands of the TSS-500 bp-TSS of genes that are significantly up-regulated (FC > 1.5) (up) or for a same number of genes (104) which RNA levels are not modified (ctrl) in shTop1 versus shCtrl HCT116 cells (transcriptomic data from [65]). Differences between the scores of the two groups were tested with a Wilcoxon rank sum test of which p-values are reported on the graphs. (C-D) Same analysis performed in the positive (**C**) or negative (**D**) strands of the promoter sequences of up-regulated and same number of control genes (123) in A549 cells (transcriptomic data from [14])

G4 structures and human TOP1 can activate or repress cellular gene transcription. In the present study, we observed a repression of HIV-1 basal transcription by both G4 and TOP1 and probably by a complex formed between these two elements. We wondered if this complex could also be involved in transcriptional repression of cellular genes. To test this hypothesis, we selected genes significantly activated under conditions of *Top1* silencing. This selection was performed on transcriptomic data obtained in two different cell lines, A549 [14] and HCT116 [65]. Cellular genes, whose RNA levels are not modified after *Top1* silencing, were selected as a control population. On these two populations, we used the G4hunter algorithm [27] to predict the presence of PQS in the positive and negative strands of the promoter regulating these genes (1–500 bp upstream of the TSS). Using a window of 25 nucleotides and a threshold score of 1.7, this predictive analysis revealed a significant enrichment of PQS in the promoters of TOP1 repressed genes (Fig. 7). This enrichment is observed in the two studied cell lines and is restricted to the positive strand of the promoters. Interestingly, no enrichment is observed in the promoters of TOP1 activated genes (Additional File 4: Fig S4), which strongly suggests that G4 structures participate in TOP1-dependent repression of HIV-1 promoter activity.

## Discussion

This manuscript presents a new role of human TOP1 as repressor of HIV-1 LTR promoter activity. This repression is observed in two cell lines (HeLa and J-Lat) and with different genetic constructs allowing to quantify the transcriptional activity of this viral promoter. In HeLa LTR Luciferase, J-Lat A1 and J-Lat 10.6 cells, *Top1* silencing by shRNA induces a reactivation of HIV-1 promoter activity, as reported by mRNA and protein levels of the reporter genes (Figs. 1, 2). In J-Lat A1 cells, different clones obtained after CRISPR/Cas9 edition of *Top1* gene, also show a significant increase in transcription initiated at HIV-1 promoter (Fig. 2). In both cells and with both strategies, the increased level of transcription is not as pronounced as the one measured in the presence of a usual inducer of HIV-1 promoter or after the expression of Tat. This limited reactivation could result from two elements. First, a residual level of TOP1 protein is observed in the cells after shRNA silencing or CRISPR/Cas9 edition of *Top1* gene which could continue to repress the viral promoter (Figs. 1, 2). Second, TOP1 is also known to favor transcription elongation along several genes [11, 14, 15, 65–68] and this effect probably occurs on the transcribed *Luciferase* and *GFP* genes. This effect on transcription elongation could also explain the differences in reactivation seen between J-Lat A1 and 10.6 cells. These latter cells have a longer transcribed sequence which potentially allows TOP1 to exert its activating effect on transcription elongation minoring its repressive effect on initiation at the viral promoter.

The TOP1 repressive role identified in this study, contrasts with the known property of TOP1 as global transcriptional activator [11, 14, 15, 18, 65, 67, 68]. Different mechanisms have been proposed for this activator role, such as TOP1 effect on DNA structure around the transcription machinery [69, 70], the recruitment of the TFIIA-TFIID complex to the TATA box [71], the activation of TOP1 catalytic activity by interacting with phosphorylated RNA Pol II CTD [15] or more recently the formation of a “toposome” complex containing TOP1, TOP2A or TOP2B and MYC/MAX or MYCN/MAX transcriptional activators [18]. TOP1 repression of HIV-1 transcription could involve different parameters and mechanisms. First, TOP1 could act in concert with cellular proteins already known to repress viral transcription. Nucleolin is a good candidate due to its interaction with TOP1 [72, 73], and its ability to suppress HIV-1 transcription through its interaction with G4s present in HIV-1 promoter [58]. Second, negative DNA topology induced by the transcription process upstream of the RNA Pol II transcribing machinery favors the opening of new transcription bubbles. By resolving this negative topology, TOP1 could interfere with new transcription initiation events. A comparative map of opened double strand DNA and DNA supercoiling along HIV-1 LTR promoter in repressed and activated conditions should give us some information on the role of these parameters in TOP1-dependent transcriptional repression. Third, SMARCA4 and SPT16, two nucleosome remodelers interacting with TOP1 [16], also have the ability to repress HIV-1 transcription [74, 75]. It would be interesting to evaluate the possible synergy of repression between these remodelers and the TOP1 enzyme. Finally, transcriptomic studies also revealed an increased transcription level of several cellular genes after *Top1* gene silencing or TOP1 inhibition [14, 18, 65]. TOP1-dependent transcriptional repression is therefore not specific for HIV-1 LTR and is conserved with other cellular promoters. The research of common parameters regulating these promoters and HIV-1 LTR should be very informative to characterize this new mechanism of transcriptional repression.

Several G4s can fold within the HIV-1 LTR promoter and one of them, called LTR III represses transcription from this promoter [50, 52, 54]. Using biophysical and structural approaches, we confirmed that the LTR III sequence can fold into a G4 structure (Fig. 4C–F, Additional file 2: Fig. S2). We also designed a new series of mutations targeting different G-tracts of this sequence and associated with a lower G4hunter score (Fig. 4B). In vitro, the four designed mutated sequences have lost their ability to fold into a G4 structure, as expected from their lower G4hunter scores. In addition, when integrated in the genome of HeLa cells, three of these sequences induce a reactivation of transcription initiated at the viral promoter. This effect, already observed with other mutated sequences in a non-integrative context [50], demonstrates that the repressive property of this G4 can be observed when it is present in a chromatin environment. Interestingly, Mut3, the only mutated sequence which is not associated with a significant reactivation of transcription (Fig. 6B), is also characterized by the highest G4hunter score and the weakest CD spectra of G4 structure (Fig. 4B, C). Therefore, this study demonstrates a direct link between the G4 folding potential of a particular LTR III sequence and its ability to repress transcription, which has not been shown before in a native chromatin context.

Human TOP1 and LTR III G4 motif are both involved in HIV-1 LTR transcriptional repression. In this study, we demonstrate that these two elements are directly interacting, either in vitro using G4 pull-down and SPR assays with recombinant or native TOP1 (Fig. 5A–D), or in a native chromatin environment, using a TOP1-ChIP assay (Fig. 6C, D). In vitro and in cells, we show that this interaction is specific for the G4 structure. By SPR, no more interaction is indeed observed using the Mut1 oligonucleotide (Fig. 5D). Using G4 pull-down assays, the quantity of interacting TOP1 is largely decreased when the LTR III bait substrate contains mutations preventing G4 folding (Mut1, Mut2 and Mut4 in Fig. 5C). Conversely, LTR III Mut3, Mut5 and Mut6 DNAs, which retain some G4 folding properties (although reduced with regards of LTR III WT DNA), are still interacting with TOP1 (Fig. 5C) and one of this mutated sequence (Mut3), does not induce a significant reactivation of transcription when inserted into the LTR promoter (Fig. 6A). In HeLa LTR Luciferase cells, mutations preventing LTR III G4 folding affect TOP1 occupancy along the viral promoter, mainly decreased in the G4 containing region (enhancer region in Fig. 6D). Altogether, the results we obtained on the G4 folding capacity of various LTR III DNAs, their interaction with TOP1 (in vitro and in cells) and their effect on HIV-1 transcription (in a chromatin environment) strongly support the notion of a TOP1-G4 complex as transcriptional repressor.

Recently, another study has reported the interaction of TOP1 with a G4 present in a cellular promoter (c-myc) and the role of the formed TOP1/G4 complex in transcriptional repression [76]. Similarly, they show a specific repression of transcription by DNA TOP1 and an inhibition of TOP1 catalytic activity by G4 DNAs folded on this sequence. Results presented in both studies argue in favor of a conserved mechanisms of transcriptional repression by a TOP1/G4 complex. In our study, we show for the first time that TOP1 and G4-dependent transcriptional repression can occur in a native chromatin environment. This parameter is crucial since G4s are sensitive to nucleosome positioning and can regulate modifications or remodeling of neighbor nucleosomes [44]. In the case of the HIV-1 promoter, TOP1 could act as a mediator between the G4 present on the promoter and nucleosome remodelers or histone modifiers regulating viral transcription. Interestingly, TOP1 can interact with different G4 structures such as the HIV-1 LTR III quadruplex/duplex hybrid scaffold [54] and the more compact structure folded on the c-myc sequence [77]. Other TOP1/G4 complexes need to be studied to determine the structural parameters associated with this interaction.

Genome-wide studies of total and active TOP1 along cellular genes have suggested a functional coupling between TOP1 catalytic activity and transcription elongation performed by the RNA polymerase II machinery [15]. In the present study, we propose a new link between TOP1 catalytic activity, its interaction with G4 structures present upstream of TSS and its ability to repress transcription. The mechanisms and partners associated with this link are still under investigation. On one hand, G4 structures, such as the one folded on the HIV-1 LTR III sequence, inhibit TOP1 catalytic activity in vitro (Fig. 5E). On the other hand, TOP1 represses transcription initiated at the viral promoter and this repression requires its catalytic activity, as observed in J-Lat A1 cells (Fig. 3). In these cells, the catalytically deficient TOP1 could displace the WT enzyme from the promoter and prevent its repressive effect. More generally, we propose that G4-motifs folded at specific promoters, such as HIV-1 and c-myc, could attract TOP1 close to the TSS and favor the recruitment of other transcriptional repressors such as Nucleolin [58]. This recruitment would require the TOP1 catalytic domain, since Y723F TOP1 cannot repress transcription anymore, even if it still binds to the G4 motifs (Additional file 3: Fig. S3 and [36]).

This model agrees with published data on TOP1 activity at cellular promoters and G4 effects on transcription. Indeed, using published transcriptomic data of two different cell lines (A549 [14] and HCT116 [65]) and the G4hunter algorithm [27] to predict the presence of PQS in cellular promoters, we observed a significant enrichment of these PQS in the promoters of TOP1-repressed genes (Fig. 7) but not of TOP1 activated genes (Additional file 4: Fig. S4). This result strongly suggests the existence of a mechanism of TOP1/G4 dependent transcriptional repression and its conservation between the viral and cellular promoters.

## Conclusions

In the present manuscript, we present a new property of TOP1 as transcriptional repressor of integrated HIV-1 promoter. We show that this enzyme interacts with a G4 structure present in the viral promoter and that this interaction is associated with a repression of its activity. G4 motifs being conserved between LTR sequences of several retroviruses [49, 56], it will be interesting to evaluate the role of TOP1 in the transcription of their genomes. In the case of HIV-1, G4s also impact other steps of its replication like reverse transcription [51, 78]. Therefore, the major roles of TOP1 and G4s in HIV-1 replication and their functional and physical interaction demonstrated in this study suggest that these two elements could be targeted at the same time in new anti-HIV treatments, similarly as recently proposed in anti-cancer strategies [79].

## Methods

### Oligonucleotides, plasmids, antibodies

#### Oligonucleotides

Desalted oligonucleotides, used for cloning and G4 pull-down assays and HPSF purified oligonucleotides used for structural studies were purchased from MWG-Eurofins. Oligonucleotides used in qPCR and ChIP assays were purchased from IDT. A list of primers used in this study is given in Additional file 5: Table S1.

#### Plasmids

The detailed sequences of all used plasmids are available upon request. pLKO1 plasmids targeting *Top1*, *Top2A* and *Top2B* genes were obtained according to Addgene Plasmid 10,878 protocol [80], using couples of primers described in Additional file 5: Table S1. shRNA sequences were selected using the MISSION shRNA Sigma-Aldrich website. plentiCRISPRv2 plasmids targeting *Top1*, *Top2A* and *Top2B* genes were obtained using the Addgene Plasmid 52,961 protocol [81], using couples of primers described in Additional file 5: Table S1. Guide RNAs were designed using TEFOR’s CRISPR/Cas9 assistant website and target codons 3, 51 and 22 of *Top1*, *Top2A* and *Top2B* coding sequence, respectively.

The pTRIP-E3-Flag-TOP1 plasmid is derived from a pTRIP ΔU3 EF1α-H10-SUMO2 from J. Seeler (Institut Pasteur, Paris, France). Briefly, this construct contains the EF1α promoter (MluI-NsiI), and sequences coding for the Flag peptide (NsiI-BamHI), an optimized 93 amino acids Auxin Induced Degron Tag (BamHI-SalI) and the human Topoisomerase1 (SalI-*Acc65I*). Topoisomerase 1 coding sequence was inserted as two consecutive segments (SalI-XbaI and XbaI-Acc65I) obtained by PCR performed on a pCDNA3-WT-TOP1 plasmid (gift of Y Pommier, NIH, USA) using Top1-Sal-For/Top1-XbaI-Rev and Top1-XbaI-For/Top1-Acc65I-Rev primer couples. Y723F mutation was introduced by subcloning a PCR segment obtained on a pCMV-Y723F-Top1 plasmid (gift of L. Yilun, COH, CA, USA) using Top1-XbaI-For/Top1-Acc65I-Rev primers.

The pFastBac1-HisTop1 plasmid was obtained by PCR amplification of the WT human Top1 cDNA, cloning in the pET28a plasmid (to insert the N-terminal His-tag) and subsequent cloning in pFastBac1 plasmid. More details about cloning procedures are available upon request to the authors.

All constructed plasmids were verified by sequencing performed by Eurofins-Genomics (http://www.eurofinscochin.com).

#### Antibodies

Anti-Top1 (ab109374) was used for ChIP assay. Anti-Top1 (ab85038), anti-Top2A (BD611326), anti-Top2B (BD611492), anti-Tubulin (Sigma DM1A), anti-FlagM2-HRP (sigma 8592) were used for western blotting.

### Cell culture, transfection, flow cytometry analysis and Luciferase assay

#### Cells

J-Lat A1, J-Lat 10.6 and Jurkat E6-1 cells were obtained from NIH AIDS reagents program and cultured in RPMI-1640 GlutaMax media supplemented with 10% fetal bovine serum (FBS) and 1X penicillin/streptomycin (Gibco). HeLa LTR-luciferase (HeLa LTR-luc) cells are a cellular clone derived from clone 9 HeLa Flp-In™ cells; their genome contains a unique copy of the *Luciferase* gene under the control of HIV LTR sequence [62]. HeLa LTR-luc and HEK-293T cells were cultured in DMEM-GlutaMAX media supplemented with 10% fetal bovine serum (FBS) and 1X penicillin/streptomycin (Gibco). All cells were cultured at 37 °C with 5% CO_2_.

#### Transfection

HEK-293T and HeLa LTR-luc cells transient transfections were performed using X-tremeGENE 9 DNA transfection reagent (Roche) according to the manufacturer’s protocol. J-Lat A1 were transfected using the Cell Line Nucleofector™ Kit V (Lonza).

#### Flow cytometry analysis

J-Lat A1 cells were fixed using 1% paraformaldehyde and GFP expression was quantified on a Flow Cytometer Accuri C6.

#### Luciferase activity

Luciferase activity of HeLa LTR-Luc cells was measured using the Luciferase Assay System kit (Promega) according to the manufacturer instructions and quantified on a FLUOstar OPTIMA (BMG LABTECH) (Fig. 1) or a CLARIOstar (BMG LABTECH) (Fig. 6).

### shRNA pLKO.1 lentiviral vectors

#### Preparation of pLKO.1 lentiviral vectors

3 × 10^6^, 293 T cells were transfected by 3 μg of pLKO.1 lentiviral plasmid (Addgene 10878) expressing the selected shRNA sequence, 2.25 μg of HIV gag-pol expressing plasmid (pL881) and 0.75 μg of VSV G expressing plasmid (pL280), using 18 μl of X-tremeGENE 9 DNA transfection reagent (Roche). Cell media was replaced by fresh media 24 h post transfection, and the media containing the shRNAs lentiviral vectors was collected 48 h post transfection, filtered with 0.45 µM filters and kept frozen at − 80 °C.

#### Transduction by lentiviral vectors

2 × 10^6^ J-Lat A1, J-Lat 10.6 or HeLa LTR Luciferase cells were transduced with 100 µL or 300 µL of shRNAs lentiviral vector. After 3 h of incubation, cells were washed twice with 1 mL PBS and resuspended in 2 mL of RMPI media containing 10% FBS and 1X penicillin/streptavidin. 24 h post transduction, cells were incubated with 1 µg/mL puromycin (Gibco). Down regulation of the selected proteins was analyzed by SDS-PAGE and western blot using corresponding antibodies.

### Generation of CRISPR J-Lat A1 cell lines

CRISPR/Cas9 edition of *Top1*, *Top2A* and *Top2B* genes in J-LatA1 cells was performed by transfection of these cells by lentiCRISPRv2 plasmids expressing a guide RNA targeting these genes (for more details, see “Plasmid construction” section). After transfection, cells were cultured during 8 days in the presence of 1 µg/ml of Puromycin. Individual clones were obtained from the bulk population of CRIPSR/Cas9 edited cells. The effect on the expression of target genes in bulk cells and individual clones was checked by SDS-PAGE and western blot of whole cell extracts.

### RNA extraction and quantification by quantitative PCR

Total RNA from J-Lat A1, J-Lat 10.6 or HeLa LTR luc cells was extracted using RNAeasy and RNAase-free, DNase kits (Qiagen). cDNA synthesis was carried out on 1 µg of DNA-free RNA using High-Capacity cDNA Reverse Transcription kit (Applied Biosystems) and random primers. Triplicated cDNA samples were analyzed by quantitative PCR using SyberGreen 2X master mix (Roche). The relative abundance of target mRNA was normalized using GAPDH as an endogenous control. Quantification of gene expression was determined using the − 2^ΔΔCt^ method. All the experiments were performed in triplicates and were repeated at least three times. qPCR quantifications comply with the MIQE Guidelines.

### Biophysical analysis of G4 folded oligonucleotides

4 µM oligonucleotide (final strand concentration) were dissolved in pH 7.2 20 mM cacodylate buffer, supplemented with 100 mM KCl, heated at 95 °C for 5 min, then slowly cooled to room temperature during 2 h.

#### Circular dichroism (CD)

CD spectra were recorded on a JASCO J-1500 spectropolarimeter at 20 °C with 2 nm bandwidth, 220–330 nm wavelength range, 100 nm/min scan speed and 0.5 nm wavelength step settings. Spectra were averaged from three scans.

#### UV/Vis difference spectra and melting/annealing experiments

UV/Vis spectra were recorded on a UVIKON spectrometer equipped with a Julabo temperature controller. Temperature was recorded by an in-cell temperature probe. Thermal difference spectra (TDS) were obtained by subtracting the UV/Vis absorbance spectra (220–320 nm wavelength range) at 5 °C from spectra at 85 °C. Isothermal difference spectra (IDS) were obtained by subtracting the UV/Vis spectra without and with potassium [82]. UV-melting/annealing processes were monitored by recording the absorbance at 295 nm between 5 and 85 °C with a 0.2 °C/min temperature changing rate. Mid-transition temperature (*T*_*m*_) was calculated by using Boltzmann function to fit the melting and annealing curves.

#### ThT assay

Oligonucleotide was diluted to 1.0 μM in the same buffer, incubated with 0.5 μM ThT (3,6-dimethyl-2-(4-dimethylaminophenyl) benzo-thiazolium bromide, 95%, Sigma-Aldrich) at 20 °C for 30 min. Fluorescence emission spectra in 450–650 nm wavelength range after excitation at 425 nm were collected on a FluoroMax-4 spectrofluorometer at 20 °C. Maximum fluorescence intensity at 490 nm was extracted to analyze the fluorescence enhancement of ThT by the oligonucleotide.

#### ^1^H NMR

100 μM renatured oligonucleotides were prepared in 20 mM pH 7.0 KPi buffer (total potassium was complemented by KCl to 100 mM) with 10% (v/v) D_2_O. 1D ^1^H NMR spectra were recorded on a Bruker 400 MHz spectrometer at 20 °C. Jump and return pulse program was used in recording proton spectra and suppressing the water signal. Scan number for each sample was 2048.

### G4 pull-down assays

G4 pull-down assays were performed on Jurkat whole cell extracts or purified recombinant TOP1. Jurkat whole cell extracts were prepared using a Lysis buffer (20 mM Tris–HCl pH 7.5; 150 mM NaCl, 1% Igepal, 1X protease inhibitor cocktail) (1.4 mL buffer per 40 × 10^6^ cells). Lysed cells were centrifuged (25 min, 4 °C, 16,000*g*) and the supernatant was collected and quantified for protein concentration. Recombinant TOP1 used in these assays was a kind gift of Y. Pommier [83].

For G4 pull-down assays, selected 3′-BITEG labelled oligonucleotides (Additional file 5: Table S1) were folded in 10 mM Tris pH 7.5, 100 mM KCl, 0.1 mM EDTA, with a 5 min 95 °C denaturation followed by a slow cool down (2 °C/min). High-affinity streptavidin magnetic beads (Pierce, 88,817) were incubated in a blocking buffer (10 mM Tris–HCl pH 7.5; 100 mM KCl; 0.1 mM EDTA; 1 mM DTT; 0.01% Triton X-100; 0.1% BSA; 25ug/mL polydIdC) prior to be incubated with the G4-folded oligonucleotides (10 μg oligonucleotides/40 µL streptavidin magnetic beads) during 90 min at 4 °C. After two washes in the cell Lysis buffer (to remove unbound oligonucleotide), the beads were incubated with whole cell extract (500 µg) or recombinant TOP1 (250 ng) during 60 min at 4 °C. Beads were then washed with Lysis buffer containing increasing KCl concentrations (200–800 mM) and retained proteins were eluted from the beads by a 5 min incubation at 95 °C in 2X SDS-PAGE loading buffer. Eluted proteins were analyzed by SDS-PAGE and western blot analysis.

### Production and purification of an N-terminal tagged human DNA TOP1 (called His-TOP1)

Expression of His-TOP1 was performed in Sf9 cell as described in [84]. Typically, 3 × 10^9^ cells were centrifuged and mixed with 15 × 10^9^ baculovirus expressing this protein (MOI of five). After one-hour incubation at room temperature, infected cells were transferred to a 1-Liter spinner and the volume was adjusted to 1 L with the expression medium supplemented with gentamicin (50 µg/ml). Protein expression was carried out for 3 days before the cells were harvested and centrifuged 30 min at 4000 rpm à 4 °C. Nuclear extracts (NE) were made from the cell pellet as described in [85]. His-TOP1 was purified from these NE following a double chromatography purification protocol. First, 10 mg of SF9 nuclear extracts (NE) were applied to 5 ml TALON^®^ Metal Affinity Resin (TaKaRa) using a batch/gravity-flow column purification procedure at 4 °C. Washing of the resin was performed using the NE buffer with 5 mM imidazole and different concentrations of KCl (150, 250, 500 and 150 mM). The resin-bound TOP1 was eluted with NE buffer complemented with 150 mM KCl and 150 mM imidazole and dialyzed against NE buffer complemented with 150 mM KCl and 1 mM DTT. cOmplete™, EDTA-free Protease Inhibitor Cocktail (Merck) was present during all steps of this first purification procedure. The second step of purification was performed by size-exclusion chromatography on a HiLoad 16/60 Superdex 200 column (GE Healthcare) equilibrated in 150 mM KCl, 20 mM Tris pH7.9, 0.2 mM EDTA, 1 mM DTT, 10% Glycerol. Selected fractions were enriched in Glycerol (20% final), aliquoted and kept at − 80 °C. Quality control of the purified protein was performed according to a previously published approach [86] following the ARBRE-MOBIEU/P4EU guidelines (https://arbre-mobieu.eu/guidelines-on-protein-quality-control/).

### Surface plasmon resonance (SPR) binding assays

His-TOP1 used in these assays was prepared as desbribed above. The SPR experiments were performed at 25 °C with a Biacore T200 apparatus (Cytiva). His-TOP1 prepared at 60 nM in 10 mM sodium acetate buffer (pH 7) was immobilized (3550 RU) on a CM5 sensor chip (Cytiva) according to the manufacturer’s instructions. A flow cell left blank was used for double referencing of the sensorgrams. The DNA samples were prepared in 10 mM HEPES buffer, pH 7.4 at 22 °C, containing 50 mM potassium chloride and 0.05% Tween-20 (running buffer). Before injected them they were denatured 4 min at 90 °C and left at room temperature for at least 10 min. The experiments were performed using the single cycle kinetics (SCK) method [87], which consists in injecting the samples at successive increasing concentrations with no regeneration step between each injection. The DNA samples were injected in duplicate for 1 min at 25 µL/min over the target. The regeneration of the TOP1 functionalized surface was achieved with a 30 s pulse of 10 mM lithium hydroxide.

### Chromatin immunoprecipitation (ChIP) assays

ChIP assays were performed using an homemade ChIP protocol derived from [88]. Briefly, 10 × 10^6^ J-Lat A1 or HeLa LTR cells, transduced seven days before by the SCR or *Top1* shRNA vectors, and diluted at 1 × 10^6^ cells per ml, are fixed with formaldehyde (1%, 10 min). This reaction is quenched with 25 mM glycine and cross-linked samples are sonicated 3 times 30 s ON/30 s OFF (Bioruptor Diagenode). For immunoprecipitation, 30 µg of chromatin extracts are incubated overnight with 2 µg of TOP1 antibody (Ab109874) or rabbit IgG (C15410206, Diagenode). The following day, the immunoprecipitated extracts are incubated during 2 h with 45 μl protein A magnetic beads, previously saturated with 1% BSA and 100 ug/ml sonicated pUC18. Immunoprecipitated samples are then washed with buffer of different salt and detergent composition and the interacting chromatin is eluted. Interacting and input chromatin are reverse cross-linked and digested by proteinase K and corresponding DNAs are recovered by successive phenol/chloroform-chloroform extractions and ethanol precipitation. Interacting and input DNA fragments are quantified by qPCR using SyberGreen master mix (Roche) and specific sets of primers (Additional file 5: Table S1). qPCR quantifications procedure complies with the MIQE Guidelines.

### DNA relaxation assays by recombinant TOP1

Recombinant TOP1 used in these assays was a kind gift of Y. Pommier [83]. TOP1 catalytic activity was quantified by relaxation of a pBR322 plasmid. 200 ng of plasmid was incubated with 70 nM TOP1 in 10 mM Tris–HCl (pH 8.8), 0.1 mM EDTA, 15 µg/ml bovine serum albumin, 50 mM KCl, during 30 min at 37 °C. G4 folded oligonucleotides (LTR III WT, Mut1, Mut2, Mut3 and Mut4, 0.1 to 10 µM) were added to the reaction assay to evaluate their effect on DNA relaxation. Reaction was stopped by addition of SDS (0.5% final) and 1µg of proteinase K and an additional incubation of 30 min at 55 °C. Supercoiled, relaxed and open circular forms of the plasmid were separated by electrophoretic migration on a 1% agarose gel, run during 4 h at 50 V in 0.5 X Tris–Acetate-EDTA buffer.

### Statistical analysis of experimental data

Experimental data were analyzed using a two-way ANOVA test or an unpaired Student test with GraphPad Prism 7 for Windows (GraphPad Software). Data shown are mean ± s.d. of three or more independent experiments. *p < 0.05; **p < 0.01; ***p < 0.001; ****p < 0.0001; ns: not significant.

### Transcriptomic data analysis

Micro-array data from HCT-116 (GSE7161, [65]) or A549 (GSE52931, [14]) cells were loaded from the GEO (https://www.ncbi.nlm.nih.gov/geo/) database. Differential expression analyses were performed in R 3.4.4 with the limma package on the log2 transformed data. A subset of genes was selected as up-regulated genes in the siTop1 condition if their RNA levels fold change (siTop1/siCtrl) > 1.5 and the corresponding fdr adjusted p-values < 0.05. The same number of genes which RNA levels were not significantly modified were considered as controls.

### Prediction of G4 structures

G4 structures were predicted for the sets of up-regulated or control genes (see transcriptomic data analysis). The TSSs of the genes were recovered in R using the biomaRt package with ensembl annotations. Fasta format files of sequences corresponding to TSS-500 bp-TSS regions were generated from these sets of genes using awk and betools. The strand information of the TSSs was considered to compute the coordinates of the TSSs-500 bp-TSS. These sequences were then used with the G4Hunter program (https://g4hunterapps.shinyapps.io/G4HunterMultiFastaSeeker/) with a window size of 25 nucleotides and a score threshold > 1.7. All graphs and statistical tests were performed in R 3.4.4.

## Supplementary Information

**Additional file 1: Figure S1.** DNA topoisomerases shRNA silencing and CRISPR/Cas9 edition in J-Lat A1 cells. Representative western blots and mRNA quantification of topoisomerases in shRNA-silenced and CRISPR-edited J-Lat A1 cells. All experiments were repeated at least 3 times.

**Additional file 2: Figure S2.** Biophysical and structural analysis of different HIV-1 LTR G4 structures. Isothermal difference spectra with and without 100 mM KCl. Thioflavinfluorescence assay. Circular dichroismspectra measured in 100 mM KCl. Oligonucleotides used in these studies are described in Fig. 4B and Addional file 5: Table S1. ^1^H NMR spectra of the G4 folded oligonucleotides in the 14.5–9.5 ppm range.

**Additional file 3: Figure S3. **Y723F mutation does not affect TOP1 interaction to HIV-1 LTR III G4 structure. Jurkat cells were transduced by a pTRIP vector expressing WT or Y723F TOP1. 8 days post transduction, total extracts of these cells were used for G4 pull-down assays, similarly as in Fig. 5B, C. This experiment was repeated at least 3 times.

**Additional file 4: Figure S4.** No G4 enrichment is observed in the positive or negative strands of TOP1 activated genes of HCT116 cells. G4 predictions in the promoter sequence of human genes repressed by Top1. Boxplots of G4 maximum scoresin positive or negative strand at the TSS-500 bp-TSS of genes that are significantly down-regulated or for a same number of genes which RNA levels are not modified in shTop1 versus shCtrl HCT116 cells.

**Additional file 5: Table S1.** Sequence of the oligonucleotides used in this study.

## Data Availability

All data generated or analyzed during this study are included in this published article and its additional files.

## Abbreviations

ChIP: Chromatin immunoprecipitation
CRISPR/Cas9: Clustered Regularly Interspaced Short Palindromic Repeats associated protein 9
DNA: Deoxyribonucleic acid
DNA Topo: DNA Topoisomerases (Topos)
GAPDH: Glyceraldehyde-3-phosphate deshydrogenase
G4: Guanine quadruplex
GFP: Green Fluorescent Protein
HAART: Highly Active Antiretroviral Therapies
HeLa LTR Luciferase: Henrietta Lacks cells encoding Renilla Luciferase protein under the control of HIV-1 LTR promoter
HIV-1: Human immunodeficiency virus type 1
J-Lat A1 and J-Lat 10.6: Jurkat cells harboring a latent HIV provirus
LTR: Long terminal repeat
NMR: Nuclear Magnetic Resonance
PQS: Putative G4 forming sequence
RNA: Ribonucleic acid
RNA PolII: Ribonucleic acid polymerase II
shRNA: Short hairpin RNA
SPR: Surface Plasmon Resonance
TOP1: DNA Topoisomerase 1
TSS: Transcriptional Start Site
