# Correction to: DNA topoisomerase 1 represses HIV-1 promoter activity through its interaction with a guanine quadruplex present in the LTR sequence

**DOI:** 10.1186/s12977-023-00627-6

**Published:** 2023-07-11

**Authors:** María José Lista, AnneCaroline Jousset, Mingpan Cheng, Violaine SaintAndré, Elouan Perrot, Melissa Rodrigues, Carmelo Di Primo, Danielle Gadelle, Elenia Toccafondi, Emmanuel Segeral, Clarisse BerliozTorrent, Stéphane Emiliani, JeanLouis Mergny, Marc Lavigne

**Affiliations:** 1 Institut Cochin, INSERM, Université Paris Cité, CNRS, Paris, F-75014 France; 2 CNRS UMR 5320, INSERM U1212, ARNA, Univ. Bordeaux, IECB, Bordeaux, 33000 France; 3 Institut Pasteur, Bioinformatics and Biostatistics Hub, Université Paris Cité, Paris, 75015 France; 4 Institut Pasteur, Departement of Virology, Université Paris Cité, Paris, 75015 France; 5 Institut de Biologie Integrative de la Cellule, CNRS, Université Paris-Saclay, Cedex, 91198 Gif Sur Yvette France; 6 Laboratoire d’Optique et Biosciences, Ecole Polytech nique, CNRS, INSERM, Institut Polytechnique de Paris, Palaiseau, 91120 France; 7 Department of Infectious Diseases, School of Immunology and Microbial Sciences, King’s College London, London, UK; 8 Université de Strasbourg, CNRS UPR 9002, Architecture et réactivité de l’ARN, Strasbourg, 67000 France; 9 School of Engineering, China Pharmaceutical University, Nanjing, 211198 China

Following publication of the original article [1], we have been informed that Fig. 6 has been superimposed on itself during Production and after author proofing, therefore rendering it illegible. Figure 6 has now been replaced.
