# Supplementary figures and images for: DNA topoisomerase 1 represses HIV-1 promoter activity through its interaction with a guanine quadruplex present in the LTR sequence
Source: Retrovirology. 2023 May 30;20:10. doi: 10.1186/s12977-023-00625-8 (PMC10228017; doi:10.1186/s12977-023-00625-8)

A.

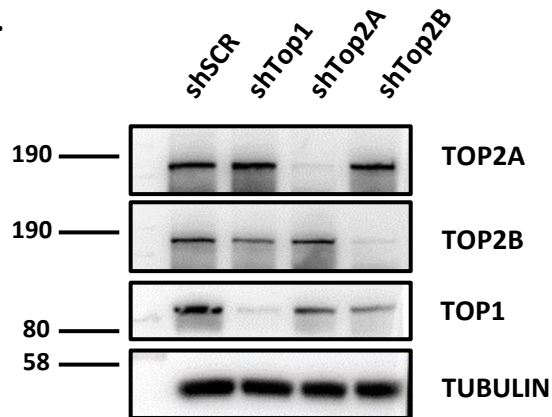

B.

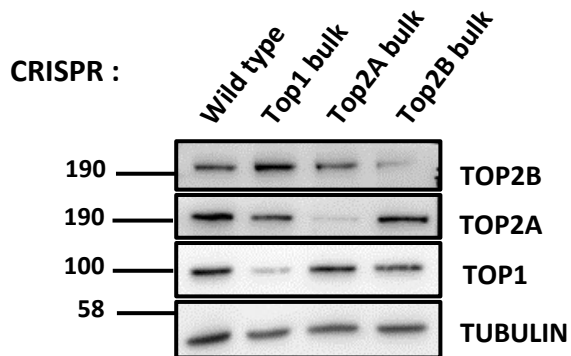

C.

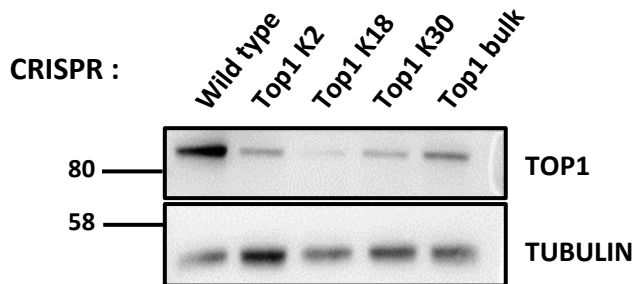

D.

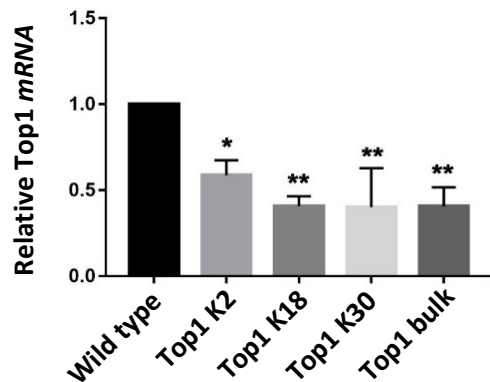

Supplement: Supplementary file 1 — Additional file 1: Figure S1. DNA topoisomerases shRNA silencing and CRISPR/Cas9 edition in J-Lat A1 cells. Representative western blots and mRNA quantification of topoisomerases in shRNA-silenced and CRISPR-edited J-Lat A1 cells. All experiments were repeated at least 3 times. [file 12977_2023_625_MOESM1_ESM.pdf]

**A.**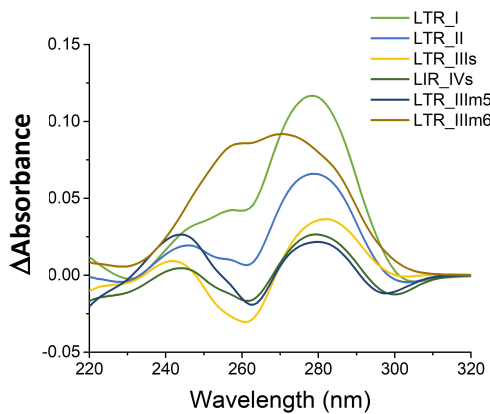**B.**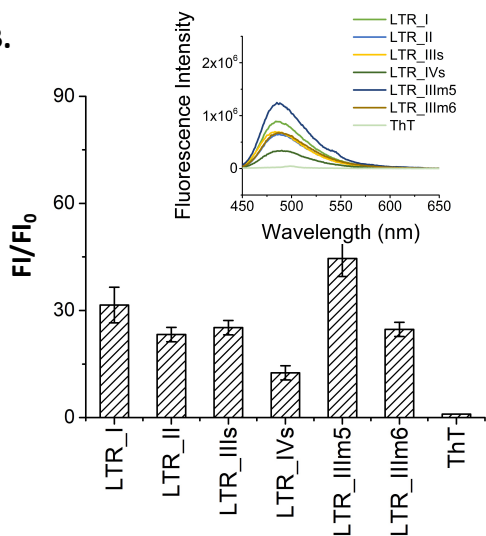**C.**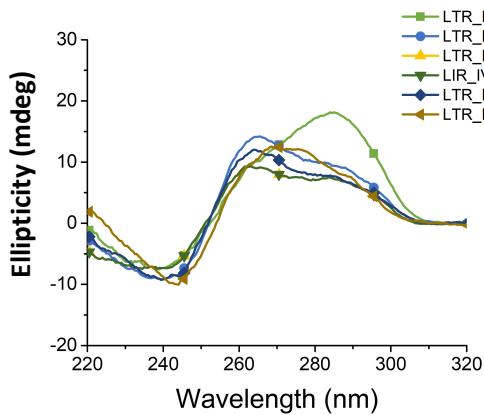**D.**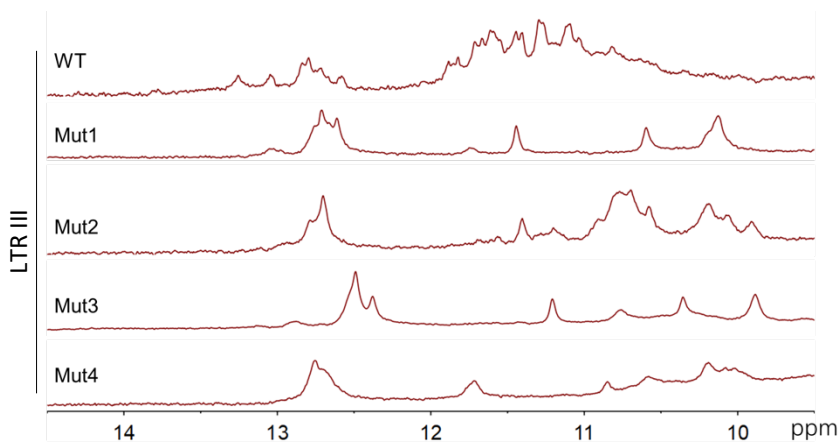

Supplement: Supplementary file 2 — Additional file 2: Figure S2. Biophysical and structural analysis of different HIV-1 LTR G4 structures. Isothermal difference spectra with and without 100 mM KCl. Thioflavinfluorescence assay. Circular dichroismspectra measured in 100 mM KCl. Oligonucleotides used in these studies are described in Fig. 4B and Addional file 5: Table S1. 1H NMR spectra of the G4 folded oligonucleotides in the 14.5–9.5 ppm range. [file 12977_2023_625_MOESM2_ESM.pdf]

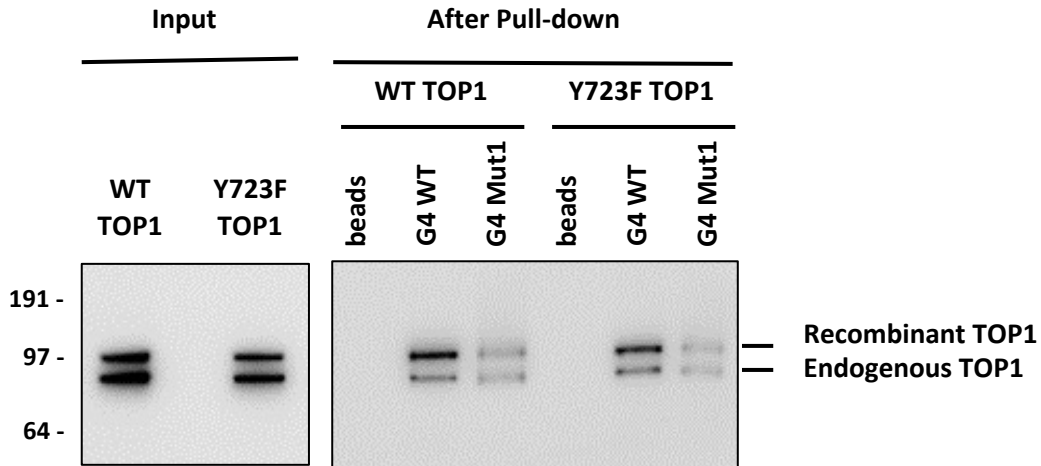

Supplement: Supplementary file 3 — Additional file 3: Figure S3. Y723F mutation does not affect TOP1 interaction to HIV-1 LTR III G4 structure. Jurkat cells were transduced by a pTRIP vector expressing WT or Y723F TOP1. 8 days post transduction, total extracts of these cells were used for G4 pull-down assays, similarly as in Fig. 5B, C. This experiment was repeated at least 3 times. [file 12977_2023_625_MOESM3_ESM.pdf]

**A. Positive strand**

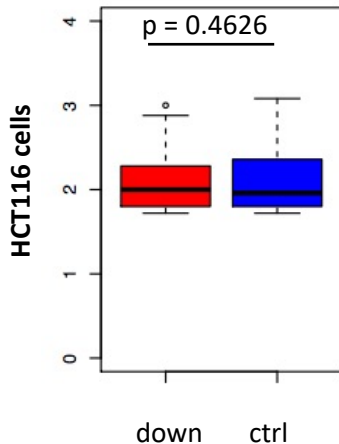

**B. Negative strand**

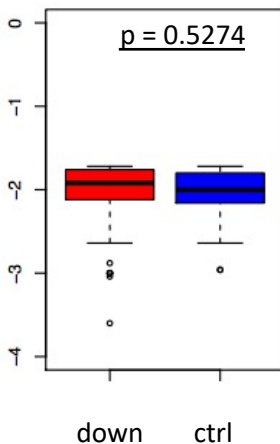

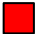 Down-regulated genes  
in Top1 silenced cells

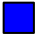 Control genes

Supplement: Supplementary file 4 — Additional file 4: Figure S4. No G4 enrichment is observed in the positive or negative strands of TOP1 activated genes of HCT116 cells. G4 predictions in the promoter sequence of human genes repressed by Top1. Boxplots of G4 maximum scoresin positive or negative strand at the TSS-500 bp-TSS of genes that are significantly down-regulated or for a same number of genes which RNA levels are not modified in shTop1 versus shCtrl HCT116 cells. [file 12977_2023_625_MOESM4_ESM.pdf]
