# Supplementary material for: DNA topoisomerase 1 represses HIV-1 promoter activity through its interaction with a guanine quadruplex present in the LTR sequence
Source: Retrovirology. 2023 May 30;20:10. doi: 10.1186/s12977-023-00625-8 (PMC10228017; doi:10.1186/s12977-023-00625-8)
Supplement: Supplementary file 5 — Additional file 5: Table S1. Sequence of the oligonucleotides used in this study. [file 12977_2023_625_MOESM5_ESM.pdf]

Supplementary Table

Oligonucleotides used for cloning in pLKO.1 (shRNA expression vectors)
ShTop1 F: 5' CCGGTGAAGGGCGAGTGAATCTAAGCTCGAGCTTAGATTCACTCGCCCTTCATTTTTG 3'
ShTop1 R : 5' AATTCAAAAATGAAGGGCGAGTGAATCTAAG CTCGAGCTTAGATTCACTCGCCCTTCA 3'
ShTop2A F: 5'-CCGGGCCCAAGTGTTCTTTAGCTTTCTCGAGAAAGCTAAAGAACACTTGGGCTTTTTG-3'
ShTop2A R: 5'-AATTCAAAAAGCCCAAGTGTTCTTTAGCTTTCTCGAGAAAGCTAAAGAACACTTGGGC 3'
ShTop2B F: 5'-CCGGTGATGATGTAATTGACGGTTTCTCGAGAAACCGTCAATTACATCATCATTTTTG-3'
ShTop2B R: 5'-AATTCAAAAATGATGATGTAATTGACGGTTTCTCGAGAAACCGTCAATTACATCATCA-3'

Oligonucleotides used for cloning in plentiCRISPRV2
CRISPR Top1 F: 5' CACCGCCTCCGGGCCGACATGAGTG 3'
CRISPR Top1 R: 5' AAACCACTCATGTCGGCCCGGAGGC 3'
CRISPR Top2A F: 5' CACCGCTCCGCCCAGACACCTACAT 3'
CRISPR Top2A R: 5' AAACATGTAGGTGTCTGGGCGGAGC 3'
CRISPR Top2B F: 5' CACCGCGGCAACGGGGCACTGACCT 3'
CRISPR Top2B R: 5' AAACAGGTCAGTGCCCCGTTGCCGC 3'

Oligonucleotides used for RT-qPCR
Luc 3' F: 5' TGGCAGGTCTTCCCGACGAT 3'
Luc 3' R: 5' GGCGACGTAATCCACGATCTCT 3'
GAPDH F: 5' TGCACCACCAACTGCTTAGC 3'
GAPDH R: 5' GCATGGACTGTGGTCATGAG 3'
Top1 F: 5'-GATGAACCTGAAGATGATGGC-3'
Top1 R: 5'-CTTCTTCCCACCATTTCCAC-3'
Top2B F: 5'-CTGCTTCTGTTTTTTCACCATC-3'
Top2B R: 5'-GCTTTCCTTTTCTTTGCCCC-3'

Oligonucleotides used for ChIP
Nuc0 S: 5'-ACAAGGCTACTTCCCTGAT-3'
Nuc0 AS: 5'-TGGCTCAACTGGTACTAGCT-3'
Enhancer S: 5'-AGAGAGAAGTGTTAGAGTGGAGGTT-3'
Enhancer AS: 5'-GCTTATATGCAGGATCTGA-3'
Nuc1 S: 5'CAGCTGCTTTTTGCCTGTACTG 3'
Nuc1 AS: 5' TCCACACTGACTAAAAGGGTCTGA 3'
GFP S: 5'GCATCGACTTCAAGGAGGAC 3'
GFP AS: 5'ACCTTGATGCCGTTCTTCTG 3'
Luc coding S: 5'AAGAGATACGCCCTGGTTCCT-3'
Luc coding AS: 5'CGGTAGGCTGCGAAATGTTCA-3'

Primers E3 constructions
Top1-Sal-For: gcgcGTCGACagtggggaccacctccac
Top1-Acc65I-Rev: gcgcGGTACCctaaaactcatagtcttcatcag
Top1-XbaI-For: catgcttaaccctagtTCTAGAatcaagggtgagaagg
Top1-XbaI-Rev: ccttctcacccttgatTCTAGAactagggttaagcatg

Oligonucleotides used for pulldown assays
LTR I: 5'-GGGACTTTCCGCTGGGGACTTTCCAGGGAGGCGTGGCCTGGG Biotin TEG-3'
LTR II: 5'-TGGGGACTTTCCAGGGAGGCGTGGCCTGGGCGGG-Biotin TEG-3'
LTR III: 5'-AGGGAGGCGTGGCCTGGGCGGGACTGGGGAGTGGC-Biotin TEG-3'
LTR III Mut1: 5'-AGCGAGACGTGGCCTGAGCGTGACTGGCGAGTGGC-Biotin TEG-3'
LTR III Mut2: 5'-AGCGAGACGTGGCCTGAGCGTGACTGGGGAGTGGC-Biotin TEG-3'
LTR III Mut3: 5'-AGCGAGACGTGGCCTGGGCGGGACTGGCGAGTGGC-Biotin TEG-3'
LTR III Mut4: 5'-AGGGAGGCGTGGCCTGAGCGTGACTGGCGAGTGGC-Biotin TEG-3'
LTR III Mut5: 5'-AGGGAGGCATGGACTGGGTGGGATTGGGGAGTGGC-Biotin TEG-3'
LTR III Mut6: 5'-AGCGAGACATGGACTGGGTGGGATTGGCGAGTGGC-Biotin TEG-3'
LTR III short: 5'-AGGGAGGCGTGGCCTGGGCGGGACTGGGGA-Biotin TEG-3'
LTR IV short: 5'--TGGCCTGGGCGGGACTGGGGAGTGGT-Biotin TEG-3'
c-myc: 5'-AGGGAGGCATGGACTGGGTGGGATTGGGGAGTGGC-Biotin TEG-3'

Oligonucleotides used for topological assays and biophyscial studies
Identical sequence as oligonucleotides used for pull down assays, without TEG at the 3'ends
